# Morphologic Characteristic of Coronary Artery Disease, with Emphasis on Thromboses, in Patients Younger Than 40 Years of Age

**DOI:** 10.4061/2010/628247

**Published:** 2009-11-05

**Authors:** Fabio Tavora, Ling Li, Mary Ripple, David Fowler, Allen Burke

**Affiliations:** ^1^Deparment of Cardiovascular Pathology, Armed Forces Institute of Pathology, 6825 16th Street NW, Building 54, Washington, DC 20306, USA; ^2^Department of Pathology, University of Maryland, Room NBW46, 22 S Greene Street, Baltimore, MD 21201, USA

## Abstract

There are few pathologic descriptions of fatal coronary artery disease in the young. The morphologic characteristics of sudden coronary deaths in 47 hearts from patients younger than 40 years were studied. Numbers of plaques with necrotic cores were quantitated in each heart. Compared to 194 sudden coronary deaths >40 years, heart weight was lower, acute plaque erosions more frequent, and extent of disease less in the ≤40 years group. Plaque burden was less in hearts with erosions, and healed infarcts more common in hearts with stable plaque. The numbers of fibroatheromas increased with age until the 6th decade (*P* < .0001) as well as the proportion of total plaques that were atheromatous. Plaques in younger patients have fewer lipid-rich cores. Most thrombi show areas of organization, with layering frequent in erosions, suggesting a possible method of plaque enlargement in the absence of necrotic core formation.

## 1. Introduction

The morphologic characteristics of acute, fatal coronary thrombi in patients aged 40 years or less are not well characterized. Patients younger than 40 undergoing coronary artery bypass graft surgery have a high incidence of coronary risk factors, especially smoking; by angiography, a high proportion of these cases have left main (13.8%) and triple vessel disease (60%) [1]. A more recent autopsy study has shown that in 11 sudden coronary deaths under age 35, there were few lipid-rich plaques, and most thrombi were erosions with ongoing organization of thrombus [2]. The purpose of this study is to determine the types of thrombi, frequency of organization, and degree of inflammation in a series of premature sudden coronary deaths with underlying thrombosis. These findings should confirm previous observations in a larger number of cases and document the progression of relatively nonlipid rich plaques to lethal occlusive thrombi.

## 2. Materials and Methods

Hearts were prospectively studied in cases of sudden coronary death, with semiserial sectioning of epicardial coronary arteries. Cases were seen in consultation from a statewide medical examiner's office over a 6-year period. Noncardiac causes of death were established by postmortem toxicology. Risk factors were determined by scene investigation.

Epicardial arteries were sectioned at 5 mm intervals and all areas with grossly identified plaque submitted for histologic analysis. Hearts were weighed with 2 cm of aorta attached, and cavitary blood removed. Myocardial scarring was classified as subendocardial (<1.3 mural thickness) and transmural. Acute plaque rupture was defined as identification of site of communication of necrotic core with lumen and luminal thrombus. Acute plaque erosion was defined as mural thrombus in the absence of exposure of lipid core to lumen. 

Severe stenosis was considered to be >75% cross-sectional luminal narrowing, as determined qualitatively. Plaque burden was estimated by 3 methods: number of major epicardial arteries with severe stenosis (left main, left anterior descending/diagonals, left circumflex/marginals, right coronary artery, 0–4 scale), number of severely stenotic segments, and a score representing the percent narrowing sum of the severely stenotic segments.

Lesions underlying erosions were classified as pathologic intimal thickening, or lipid pools without necrotic core [3], or fibroatheroma, with necrotic gruel, cellular breakdown, with or without intraplaque hemorrhage. Numbers of fibroatheromas were quantitated in all hearts and also expressed as a ratio of all segments examined. Thrombus organization was defined differently for erosions and ruptures. In erosions, fresh (or acute) thrombus denoted the absence of fibrin incorporated within the fibrous cap, early organization the presence of fibrin with the smooth muscle-cell rich cap without layering, and late organization the presence of distinct fibrin layering within the smooth muscle cell rich cap. For acute ruptures, early organization denoted endothelialization over a portion of the thrombus; late organization the presence of smooth muscle cell ingrowth in a portion of the thrombus. In all cases, acute thrombus indicats the presence of at least one portion of the luminal thrombus with fibrin platelets at the surface without surface organization.

Immunohistochemistry was performed at the site of thrombus, if present. Macrophages were identified by CD68, KP-1 clone (Dako, Carpinteria, dilution 1:50), and T-lymphocytes by CD45RO (Dako, dilution 1:50). Quantitation of cellular elements was performed in the fibrous cap region by computerized morphometric measurements (IPLab SpectrumTM image processing software, Signal Analytics Corporation, Vienna, VA). Statistical analysis was performed using SAS software (Cary, NC). Comparison of two means was performed using Student's *t*-test, and of multiple categories was performed using ANOVA means table with Fisher's post hoc testing.

## 3. Results

In the study period there were 241 hearts studied. There were no significant differences between the ≤40 group and the >40 group in proportion of women, black race, smokers, diabetics, or hypertensives. (Table 1). There was no difference in body mass index; however, heart weight was significantly less in the ≤40 year group (Table 1, *P* < .0001). The culprit plaque was acute thrombus, either rupture or erosion, in 45% of patients >40 years versus 68% of ≤40 years; the difference in the rate of erosions (14 versus 36%, respectively) was highly significant (*P* = .0005). The number of epicardial arteries with ≥75% cross sectional area narrowing was greater in the >40 year group (2.0 ± .8 versus 1.8 ± 0.9 for the ≤40 year group, *P* = .3), and the number of stenotic segments significantly greater (3.9 ± 1.6) than the ≤40 group (3.2 ± 1.9, *P* = .02). Plaque burden was significantly greater in the older hearts (*P* = .004, Table 1).

**Figure 1:**
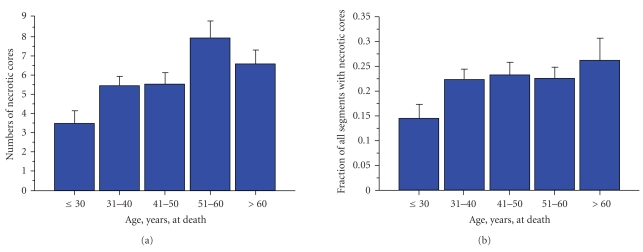
Quantitation of necrotic cores in hearts from patients dying sudden with severe coronary disease. (a) The numbers of necrotic cores increased with advancing age, reaching a maximum in the sixth decade. There was a significant increase between ≤30 years and both the 7th and 8 decades (*P* < .0001). (b) Adjusted for plaque burden (numbers of atheromatous plaques/total number studied), the number of fibroatheromas was similar across all ages, with the exception of under age 30, which demonstrated a significantly lower number. There was a significant increase between ≤30 years and the 5th decade (*P* = .04), the 6th decade (*P* = .02), and the 7th decades (*P* = .02).

The numbers of fibroatheromas increased with age until the 6th decade (Figure 1(a)). There was a significant increase between ≤30 years and both the 7th and 8 decades (*P* < .0001). The fraction of plaques with necrotic cores (fibroatheromas / total segments studied) also increased with age (Figure 1(b)); there was a significant increase between ≤30 years and the 5th decade (*P* = .04), the 6th decade (*P* = .02), and the 7th decades (*P* = .02).

There were 47 deaths ≤40 years of age. Culprit plaque was acute rupture in 15, acute erosion in 17; in the remaining 15, there were no acute thrombi. There were no differences by culprit plaque in mean age, body mass index, incidence of transmural infarct, or heart weight among the three groups (stable plaque, acute erosion, or acute plaque rupture) (Table 2). However, the number of women with acute ruptures was 0 (*P* = .004 compared to other groups); blacks were more frequent in deaths due to erosion (*P* = .04). The plaque burden was significantly less in the erosion group, as determined by number of severely stenotic epicardial arteries, number of severely stenotic segments, and estimated plaque burden. 

Of the 32 thrombi, 1 was present in the left main (plaque erosion), 19 in the left anterior descending (14 proximal, 4 mid, and 1 distal, 11 erosions and 8 ruptures), 10 in the right coronary (4 proximal and 6 mid, 5 erosions and 5 ruptures), and 2 in the left circumflex/obtuse marginal (2 ruptures).

No organization was present in the acute thrombus of 3/17 erosions and 3/15 ruptures (6/32 total, or 19%). In the remainder, there were early organization in 8 erosions and 6 ruptures and late organization in 6 erosions and 9 ruptures (Table 3). There was no correlation between organization of the plaque and presence of myocardial necrosis (Table 3). 

**Figure 2:**
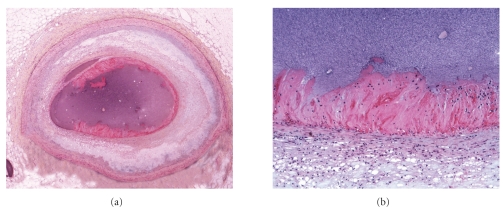
Acute erosion, without organization, Movat pentachrome. (a) Low magnification of a cross section of the left anterior descending coronary artery. The underlying intima shows lipid pools of pathologic intimal thickening with focal early atheromatous core formation. (b) Higher magnification of acute fibrin platelet thrombus, which is nonocclusive. Black material represents contrast agent inject postmortem.

**Figure 3:**
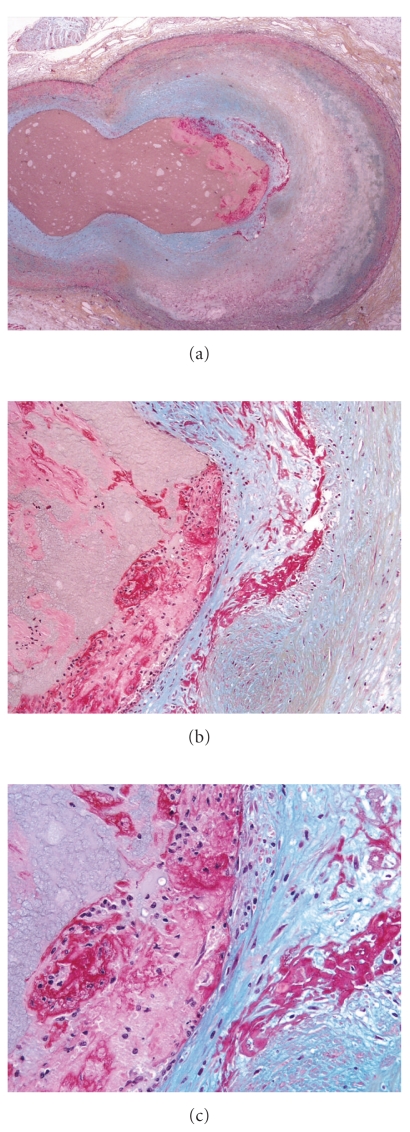
Acute erosion, with early organization, Movat pentachrome. (a) Low magnification of a cross section of the left anterior descending coronary artery, at the branch point of the first diagonal. The intimal plaque is smooth muscle cell rich without atheromatous core formation. (b) There is a nonocclusive luminal thrombus, with a single strand of fibrin incorporated in the smooth muscle cell, proteoglycan rich cap. (c) Higher magnification of (b), demonstrating the acute nonocclusive thrombus on the left, and the fibrin incorporated within the cap on the right.

**Figure 4:**
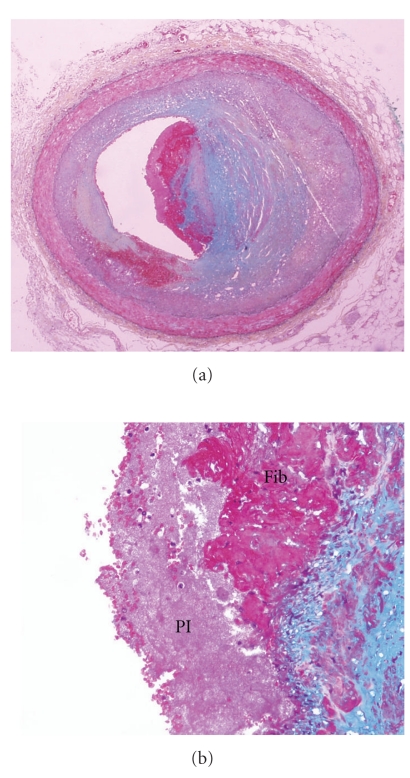
Acute erosion, with early organization, Movat pentachrome. (a) Low magnification of the left anterior descending coronary artery with severe luminal narrowing and nonocclusive thrombus. The underlying plaque is rich in smooth muscle cells, without significant lipid and no core formation. (b) Higher magnification demonstrating a single layer of fibrin in the proteoglycan-rich cap (right). The center of the photomicrograph demonstrates layering of the thrombus, with platelets (Pl) adjacent to the lumen overlying fibrin (Fib).

**Figure 5:**
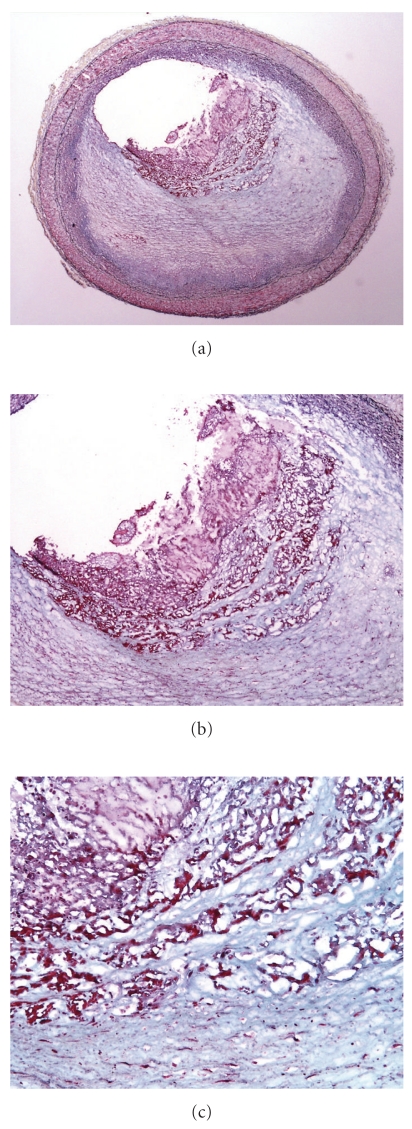
Acute erosion, with late organization, Movat pentachrome. (a) Low magnification of the left anterior descending coronary artery. The underlying plaque is rich in smooth muscle cells, without significant lipid and no core formation (b) A higher magnification of the nonocclusive eroded thrombus. (c) Multiple layers of fibrin are present in the smooth muscle cell rich cap.

**Figure 6:**
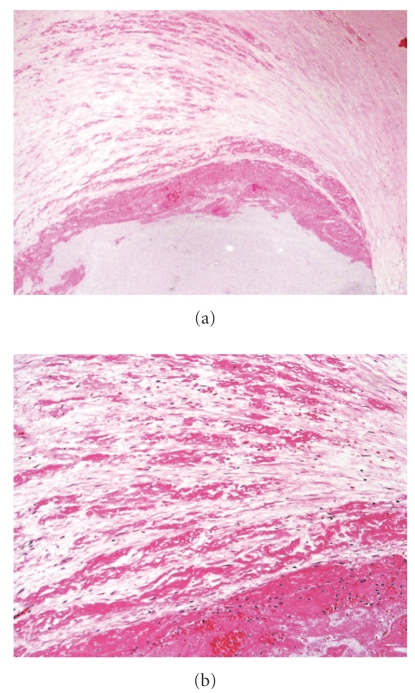
Acute erosion, with late organization. (a) Cross section of mid right coronary artery shows nonocclusive thrombus; (b) demonstrates multiple layers of fibrin within the plaque.

**Figure 7:**
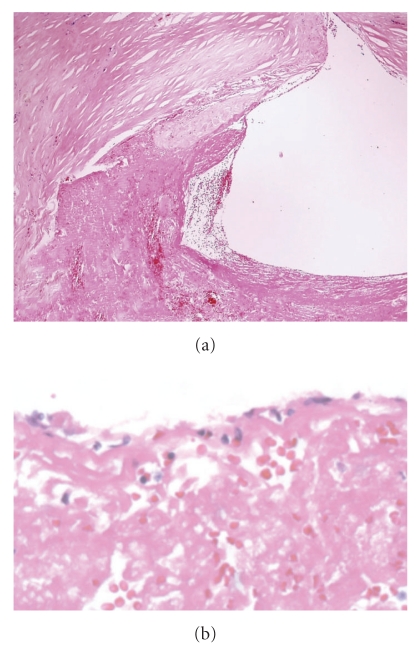
Acute plaque rupture, with early organization. (a) A disruption fibrous cap is seen above, with nonocclusive luminal thrombus. (b) There is endothelial cell ingrowth over the surface of the thrombus.

Organization of erosions progressed from acute fibrin platelet thrombus without fibrin in the underlying cap (Figure 2), to single (Figures 3 and 4) to multiple (Figures 5 and 6) layers of fibrin. Underlying the plaque erosions, the plaque was predominantly fibrous, with some extracellular lipid (pathologic intimal thickening), but no necrotic core, in 16 of 17 (Figures 3–6); a necrotic core was present in 1. In plaque ruptures, early organization consisted of endothelialization (Figure 7) and late organization smooth muscle cell ingrowth. 

Lymphocyte numbers were greater in erosions than ruptures, but without significance (*P* = .2, Table 4); there was significantly increased macrophages infiltrates in ruptures as compared to erosions (*P* = .0001, Table 4).

## 4. Discussion

The current study shows that, compared to sudden coronary deaths in older individuals, those occurring under age 40 have several differences. Most notably, there is a higher proportion of acute plaque erosions, and heart weight and overall plaque burden are less. These results are consistent with the study by Henriques de Gouveia et al. [2], who demonstrated a preponderance erosions in premature sudden coronary deaths, and of Virmani et al. [4]who demonstrated that severity of disease is less in fatal coronary disease in patients under age 30. The current study also demonstrates that heart weight is less in younger patients, which may be related to acquired disease with cumulative effects on cardiomegaly in more advanced age. Although the incidence of hypertension was not significantly higher in the older age group, the duration of the disease would likely be greater and hence the likelihood for myocardial hypertrophy.

Within the group ≤40 years, the current study showed that plaque ruptures were not found in women, which is consistent with prior reports of the so-called “protective” effect of estrogen on the formation of plaque rupture [5]. In addition, we found lesser plaque burden in patients with erosions, associated with fewer healed infarcts. The significance of these findings is uncertain but suggests that eroded thrombi may occur relatively rapidly and may cause death before extensive myocardial scarring and diffuse disease.

Acute coronary syndromes, including acute myocardial infarction and sudden coronary death, occur by either rupture of a thin fibrous cap or erosion of a deendothelialized surface in the absence of cap disruption [6, 7]. Although the mechanisms of the initiation of coronary thrombosis have been extensively studied, especially in the case of plaque rupture, [8] relatively little is known about coronary thrombus propagation and organization. It has generally been accepted that acute ST-elevation myocardial infarction results from a rapidly developing thrombus, especially in the case of plaque rupture, which is conceptualized as a dramatic event leading instantaneously to symptoms or death. However, healed coronary plaque ruptures with the deposition of fibrous tissue have been documented in detail in autopsy studies [9, 10] Furthermore, histologic analysis of thrombosuction during percutaneous intervention (PCI) for acute ST-elevation myocardial infarction has revealed that the thrombus originated days before the acute presentation of symptoms in more than one-half of patients [11]. Furthermore, an autopsy study in premature coronary thrombosis showed that 8 of 11 thrombi demonstrate organization [2]. The current study corroborates these observations, indicating that, at the time of lethal arrhythmia resulting in death, the thrombus had been ongoing in a majority of cases, with almost one-third demonstrating smooth muscle cell migration, indicating at least several days duration.

The current study demonstrates that the rate of thrombus is higher in premature sudden coronary death as compared to older individuals. The incidence of acute thrombi in sudden death is controversial, ranging from nearly all [6], to more intermediate numbers, such as 62% [12] in a series of 206 patients, 73% in a series from Mayo Clinic [13] (32% of which were erosions), and 57% [14] from an urban medical examiner. The incidence of acute thrombi decreases with comorbid conditions that may result in ventricular arrhythmias, such as hypertension [15]. Therefore, it is possible that the relatively high rate of thrombi in the current study reflects the greater rate of cardiomegaly and healed infarcts in older patients.

This study also emphasizes the need for extensive sampling of the coronary tree, in sudden death cases autopsied at a medical examiners office and hospitals, as it may be an important undervalued cause of death in young patients. Evaluation of grossly visible plaques often shows important information regarding mechanism of death. In addition, microscopic evaluation of ventricular myocardial sections may reveal microscopic areas of ischemia and other substrates for arrhythmias. 

Coronary plaques in patients dying suddenly have been shown to demonstrate relatively decreased inflammation [4]. Plaque erosions, which are overrepresented in young patients in the current study, as well as that of Henriques de Gouveia [2], show less inflammation than acute ruptures [7]. It is tempting to speculate that plaque erosions occur on relatively early stage plaque, without well-developed necrotic cores, whereas plaque ruptures represent a later stage which occur at later ages with more extensive inflammation. The gradual increase in numbers and proportions of necrotic cores in the current study supports this hypothesis. 

In conclusion, we have demonstrated that premature sudden coronary death <40 years demonstrates a relatively high numbers of acute thrombi, especially erosions, that organization is frequent, and that erosions have fewer macrophages than ruptures. These results corroborate previous observations. In addition, we have shown that premature coronary deaths demonstrate a lesser degree of cardiomegaly and healed infarcts and that the necrotic core formation is associated with increasing age. These findings may have implications for imaging of plaque components in young patients with coronary symptoms as well as corroborate an alternative method for plaque enlargement in relatively lipid-poor plaques.

## Figures and Tables

**Table 1:** Comparison of patients dying with severe coronary disease <41 and >40 years of age.

	<41 years *n* = 47	>40 years *n* = 194	*P* value
Age, range	22–40	41–78	—
% women	31	23	.2
% black	13/44	53/140	.3
BMI, SD	27.9 ± 6.4	28.4 ± 5.7	.6
% smokers	70	57	.2
% diabetic	36	24	.2
% hypertensive	29	36	.3

**Table 2:** Comparison of cardiac findings with sudden coronary death <41 years versus >40 years.

	<41 years *n* = 47	>40 years *n* = 194	*P* value
Heart weight gm SD	409 ± 94	498 ± 119	<.0001
% acute ruptures	32	31	.7
% acute erosions	36	14	.0005
% healed infarcts	40	53	.1
Number stenotic vessels	1.8 ± .9	2.0 ± .8	.3
Number stenotic segments	3.2 ± 1.9	3.9 ± 1.6	.02
Estimated plaque burden**	240	304	.004

**ANOVA means table with Fisher's post hoc test.

**Table 3:** Patient and heart characteristics, ≤40 years of age (**n** = 47), comparison of patient and cardiac features, by culprit plaque.

Characteristic	Stable *n* = 15	Rupture *n* = 15	Erosion *n* = 17	*P* value
Age, years SD	36 ± 2	37 ± 4	36 ± 5	.8
M:F	7 : 8	15 : 0	10 : 7	.004*
White:Black	11 : 3	13 : 2	8 : 9	.04*
BMI	28 9	28 5	28 4	.9
Any HMI, %	67	33	18	.0004*
Transmural HMI, %	26	7	6	.1
Heart weight, gms SD	389 ± 75	438 ± 88	401 ± 98	.1
Number stenotic arteries	2.2 ± 1.0	1.9 ± 0.9	1.5 ± 0.7	.04 stable versus erosion**
Number stenotic segments	3.9 ± 2.0	3.7 ± 1.8	2.4 ± 1.5	.02, erosion versus others**
Plaque burden	307 ± 162	288 ± 147	159 ± 111	<.01, erosion versus others***

*Chi-squared test. **ANOVA means table with Fisher's post hoc test. ***ANOVA means table with Fisher's post hoc test; *P* = .006 versus ruptures and .002 versus stable plaque.

**Table:** (a) Rate of healing, erosions versus ruptures. Focal thrombus organization.

Acute Erosions	*17*	Myocardial necrosis, *n*
No organization	3	1 (microscopic necrosis)
Early organization	8	3 (microscopic necrosis)
Late organization	6	0
Acute Ruptures	15	
No organization	3	1 (microscopic)
		1 (acute subendocardial infarction)
Early organization	6	1 (microscopic)
Late organization	9	1 (microscopic)

**Table:** (b) Quantitative data.

	Erosions	Ruptures	*P* value, erosions versus ruptures
Macrophages/mm^2^	127 ± 27	368 ± 57	*P* = .0001
Lymphocytes/mm^2^	171 ± 30	121 ± 18	*P* = .2
